# Application of the Gross Motor Function Measure-66 (GMFM-66) in Dutch clinical practice: a survey study

**DOI:** 10.1186/s12887-015-0459-8

**Published:** 2015-10-07

**Authors:** Laura WME Beckers, Caroline HG Bastiaenen

**Affiliations:** Department of Epidemiology, CAPHRI School for Public Health and Primary Care, Maastricht University, PO Box 616, 6200 MD Maastricht, The Netherlands; Department of Rehabilitation Medicine, CAPHRI School for Public Health and Primary Care, Maastricht University, PO Box 616, 6200 MD Maastricht, The Netherlands

**Keywords:** Cerebral palsy, Children, Clinical practice, Evidence based, Gross Motor Function Measure, Implementation, Knowledge translation, Motor function, Physiotherapy, Research uptake

## Abstract

**Background:**

The Gross Motor Function Measure-66 (GMFM-66) is an observational clinical measure designed to evaluate gross motor function in children with Cerebral Palsy (CP). It is a shortened version of the GMFM-88. A free computer program, the Gross Motor Ability Estimator (GMAE), is required to calculate the interval level total score of the GMFM-66. The aim of this study was to explore pediatric physiotherapists’ experiences with the GMFM-66 and application of the measure in Dutch clinical practice.

**Methods:**

An explorative cross-sectional survey study was performed. Dutch pediatric physiotherapists were invited to complete an online survey. Data-analysis merely consisted of frequency tables, cross-tabulations and data-driven qualitative analysis.

**Results:**

Fifty-six respondents were included in the analysis. In general, the therapists expressed a positive opinion on the GMFM-66, in particular regarding its user-friendly administration and benefits of the GMAE. The majority of questions revealed that therapists deviate from the guidelines provided by the manual to a greater or lesser extent though. The most worrisome finding was that 28.8 % (15/52) of the therapists calculate the total score of the GMFM-66 using the score form of the GMFM-88 instead of the GMAE.

**Discussion:**

The consequences of the high number of therapists who stated that they calculate the total score of the GMFM-66 with the GMFM-88 score form are far-reaching; it has a misleading impact on the opinion of rehabilitation teams and parents on the development of the child, on decision-making in rehabilitation, and ultimately on the development of the child.

**Conclusions:**

Information currently available on psychometric properties, motor growth curves and percentiles cannot be generalized to clinical practice in the Netherlands, as they were generated in highly controlled testing conditions, which do not hold in clinical practice.

**Electronic supplementary material:**

The online version of this article (doi:10.1186/s12887-015-0459-8) contains supplementary material, which is available to authorized users.

## Background

Evaluation of motor function is essential to monitor and adjust therapies to optimize the effect of rehabilitation of children with cerebral palsy (CP). Numerous clinical measures are available for such evaluation. In the Netherlands the Gross Motor Function Measure-66 (GMFM-66) and the original 88-item version (GMFM-88) are recommended to measure motor abilities on the activity level in children with CP [1], with GMFM-66 the more popular one given its reduced administration time.

The GMFM-66 was developed in Canada as an observational clinical measure to evaluate gross motor function in children with CP [2]. The GMFM-88 and GMFM-66 consist of respectively 88 and 66 items, divided into five categories (lying and rolling; sitting; crawling and kneeling; standing; walking, running, and jumping). Each item is scored on a four-point Likert scale. The instruments were developed for evaluative purpose. Both measures have been validated in children with CP from 5 months to 16 years of age. A 5-year old child without motor disabilities is able to reach the maximum score [2]. The total score of the GMFM-88 is calculated by a score form for all dimensions or specific dimension(s) of interest. For the GMFM-66 a free computer program, the Gross Motor Ability Estimator (GMAE), is required to calculate total scores. The advantage of the program is it can convert individual item scores into an interval level total score. The interval level was developed by Rasch analysis, based on item response theory [3].

Although the GMFM-66 is often seen as an improvement on the GMFM-88, the latter has its own strengths and should be the preferred instrument in certain situations. First, of the 22 additional items of the GMFM-88 13 belong to the dimension ‘lying and rolling’, 5 to ‘sitting’, and 4 to ‘crawling and kneeling’. Consequently, for young children and children with severe motor disabilities the GMFM-88 gives a more detailed description of their abilities and limitations. Moreover, the GMFM-88 can be administrated with shoes, ambulatory aids and/or orthoses, whereas the GMFM-66 must be administrated barefoot without aids. Although the GMFM-88 has been developed for children with CP, it is also validated for other populations, such as children with Down Syndrome and acquired brain damage. At present the GMFM-66 is only validated in children with CP. Benefits of the GMFM-66 include a reduction in time needed for administration, the possibility to assess selected items only (item maps), availability of interval-levels of the total score and confidence intervals (CI) of the total score. As stated in the manual, to define whether a change is a true change or based on measurement error, the 95 % CI’s between the two tests should be compared. If the CI’s overlap the change may be due to measurement error, but if they do not overlap it is a true change. Additionally, the GMAE provides various extra features, including standard error of measurement (SEM), motor development curves, and percentiles stratified by age and level on the Gross Motor Function Classification System (GMFCS). Item maps show which items the child has achieved and which ones he/she will likely accomplish next [2].

**Fig. 1:**
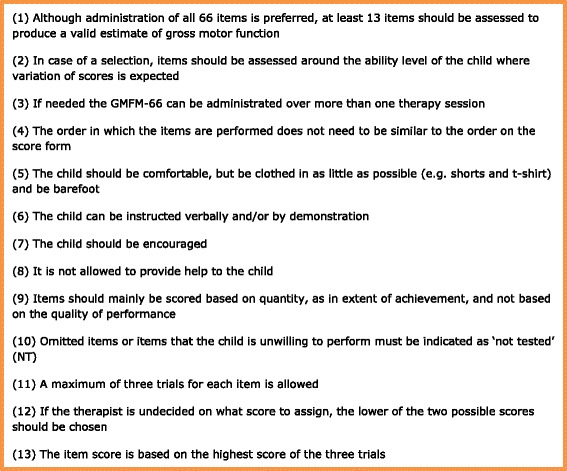
Administration guidelines GMFM-66

Both versions, the original GMFM-88 and the shortened GMFM-66, have been translated into Dutch [4–6]. For the GMFM-88 a Dutch manual is also available. For the GMFM-66 only an English manual exists. It is recommended to consult the manual during assessment, since it provides detailed item scoring guidelines in addition to more general guidelines regarding administration. The most relevant guidelines for administration of the GMFM-66 are presented in Fig. 1.

Studies evaluating the application of instruments in clinical practice are scarce, which is a limitation of evidence-based practice in (Dutch) pediatric rehabilitation. Psychometric properties are generally defined based on highly controlled assessments and results cannot be generalized to clinical practice. Furthermore, for measures that have been developed for use in clinical practice, evaluation of whether the instrument fulfills the needs of its users should take place, feasibility should be verified, and contradictions with guidelines should be pointed out. It may also be helpful to make pediatric physiotherapists in clinical practice aware of the fact that psychometric research on an instrument is focused on both the observers and the children as separate sources of variation in the received data.

Evaluation of the application of the GMFM-66 has priority due to its popularity in clinical practice. To our knowledge only one study to date discusses the experiences of therapists (*n* = 12) with the GMFM-66 regarding familiarity, confidence, and application [5]. Although this study provides some indications concerning application of the GMFM-66, additional evaluation is required. First, because of the small sample size no statements can be made regarding the application of the test in clinical practice. Furthermore, due to the selection method used, information is missing from a large group of therapists who did not attend the workshop, despite using the GMFM-66 [5]. Thorough evaluation of the experiences of a larger and more heterogeneous group of therapists will therefore add crucial information to the existing knowledge base.

The aim of this study was to evaluate the application of the GMFM-66 in Dutch clinical practice from the therapists’ perspective, by an explorative cross-sectional survey study, making use of an electronic questionnaire in a heterogeneous population.

## Methods

### Survey development

The survey used in this study was developed by reviewing the Gross Motor Function Measure (GMFM-66 & GMFM-88) User's Manual [2]. To gather information on the variety of ways in which therapists use the instrument and their motives, 52 questions were formulated covering five topics: (1) baseline characteristics, level of experience with GMFM-66 and overall impression of the instrument; (2) GMFM-66 versus GMFM-88; (3) goal and target-population; (4) administration and scoring; (5) interpretation. A combination of structured and unstructured questions was used. The survey was formatted on the software tool Formdesk to be administrated electronically and securely. Only the questions on baseline characteristics were selected as required, since missing values were preferred over terminated questionnaires. Based on a pilot-study among students of the Master Pediatric Physical Therapy of the Avans+ institute (*n* = 6), several questions were edited based on general feedback. The maximum time needed to complete the questionnaire was estimated at 15 min.

### Survey instrumentation

The target population consisted of pediatric physiotherapists in the Netherlands who had used the GMFM-66 at least once in the previous 6 months, which was checked through the first item of the questionnaire. Since registration of all pediatric physiotherapists is not available and the results were aimed to be generalizable to the whole population, recruitment was fourfold. First, members of the Dutch Association for Pediatric Physical Therapy (NVFK), consisting of approximately 1100 physiotherapists [7], were recruited by a call on the association’s website and their electronic newsletter (*n* = 1020). Second, a call was posted in the LinkedIn group ‘Pediatric Physical Therapists in the Netherlands’, which included 900 members at that time. Third, Knowledge Brokers were contacted and asked to invite all pediatric physiotherapists of their center to participate. Knowledge Brokers are health professionals intended to create connections between researchers and clinical practice to promote evidence-based decision making. The Dutch CP Knowledge Brokers collaborate by a national network, and mainly focus on implementation and application of measures. Finally, all members of a study group for (para)medical professionals working in neurorehabilitation (Studiegroep Neurorevalidatie Keypoint) were invited by a call on an invitation for a seminar. For each recruitment strategy an appropriate explanation of the research was given, where necessary including a link to the more detailed call on the NVFK website. Filling out the survey implied that the therapist agreed with participation. The survey could be exited at any time. This study does not fall under the scope of the Dutch Medical Research Involving Human Subjects Act (WMO) as no patient data were collected. Only the opinion of physiotherapists was requested by the survey, hence ethical approval was not required.

### Data analysis

Demographic characteristics of the study population were explored. Frequencies were calculated for categorical questions and measures of central tendency and variability for continuous variables. Cross-tabulations of the extent to which therapists follow the guidelines were created for two variables: whether a respondent participated in the Training GMFM and whether a respondent fulfilled the function of Knowledge Broker. Independency between ‘participation in the Training GMFM’ as well as ‘fulfilling the function of Knowledge Broker’ and use in populations other than children with CP as well as way of calculating the total score was tested by the Fisher’s exact test. Because of the explorative character of the study no correction for multiple testing was used, since type II error was preferred over type I error. Analysis of unstructured questions began by reading all responses given for each question, to get an overview of the data. The answers for each question were fragmented, coded and categorized by identifying descriptive words by a data driven approach. Additionally, patterns between answers on the various unstructured and structured questions were investigated.

## Results

### Demographics

Data were collected through a cross-sectional design from December 2013 until end of February 2014. There were 107 respondents in total, of whom 57,9 % (62 respondents) met the inclusion criterion. Fifty-six respondents who provided at least all demographic and professional information were included in the study. Table 1 provides the demographic and professional characteristics of the included respondents. Responses of a few pediatric physiotherapists in training were included in analysis. For frequency of assessment three outliers, of which one was an extreme value, were detected. These were included in the analysis though since there was no indication that these were errors. All continuous variables were found to be significantly non-normal by the Kolmogorov-Smirnov test.

**Table 1:** Demographic and professional characteristics

Variable	Frequency	Median	1st quartile	3rd quartile
Gender				
Male	6/56 (10.7 %)			
Female	50/56 (89.3 %)			
Age		39.0	29.0	53.0
Area of practice				
Primary care^a^	12/56 (21.4 %)			
Secondary care^a^	33/56 (58.9 %)			
Tertiary care^a^	14/56 (25.0 %)			
Type of qualification				
Dutch Master of pediatric physiotherapy	26/56 (46.4 %)			
No Dutch Master of pediatric physiotherapy	15/56 (26.8 %)			
Else	15/56 (26.8 %)			
Present education				
None	48/56 (85.7 %)			
Dutch Master of pediatric physiotherapy	4/56 (14.3 %)			
Else	0/56 (0.0 %)			
Years since graduation		7.0	3.0	16.0
Still in training	5/56 (8.9 %)			
Knowledge Broker^b^				
Yes	22/56 (39.3 %)			
No	33/56 (58.9 %)			
Not known	1/56 (1.8 %)			
Resources used to get competent regarding the GMFM-66				
GMFM Self-Instructional Training CD-ROM^a^	15/56 (26.8 %)			
Colleagues^a^	38/56 (67.9 %)			
English Manual^a^	25/56 (44.6 %)			
Master of pediatric physiotherapy^a^	19/56 (33.9 %)			
Training GMFM^a^	30/56 (53.6 %)			
None of the above^a^	0/56 (0.0 %)			
Else^a^	7/56 (12.5 %)			
Years of experience with the GMFM-66		6.0	3.5	10.0
Not known	9/56 (16.1 %)			
Frequency of GMFM-66 assessment (past year)		11.0	4.3	18.8

^a^Multiple answers possible

^b^The Dutch CP Knowledge Brokers collaborate by a national network and mainly focus on implementation and application of measures

### Primary analysis

Two open-ended questions focused on general opinions of the GMFM-66 and suggestions for improvement. All views of the therapists are presented in (Additional file 1: Figure S2). Seven topics were identified in which several themes recurred.

Both the implicit and explicit comments of the respondents showed their *general impression* of the GMFM-66 to be very positive. The instrument was frequently described as useful, clear and nice. For *application*, the GMFM-66 was considered useful for evaluative purposes. Regarding *content* some therapists expressed appreciation of the conciseness, while others felt the extent of the instrument is too limited. Therapists generally indicated the *assessment* of the GMFM-66 is very user friendly. However it was noted that administration is difficult in children with mental retardation or behavioral issues. A few respondents expressed that children enjoy performing the test and showing their abilities. Some therapists felt that the *scoring* of items is objective, while others reported a high level of interpretability. A common view amongst therapists was that the *GMAE* is valuable and user friendly. Percentiles, reference curves and item-maps were mentioned as useful features. Within the topic *interpretation* therapists expressed limited sensitivity to change in general and especially in young children, severely affected children and slightly affected children (ceiling effect).

Suggestions for improvement were only sparsely given by respondents and were very diverse, yet three issues were recurring. Some therapists expressed the need for a high quality instruction DVD, a more specific item scoring description was suggested in order to increase objectivity, and a version more suitable for severely affected children was requested.

Respondents were asked to explain for what reason(s) they decided to use the GMFM-66 or GMFM-88 in clinical practice. Some therapists expressed a strong preference for one instrument, usually the GMFM-66, sometimes in agreement with their team. Both limited time for assessment and the advantages of the GMAE were common general reasons for choosing the GMFM-66. Therapists mentioned the need for thorough evaluation of specific domains and interest in items only included in the GMFM-88 as motivations for using the GMFM-88. Additionally, patient specific considerations were indicated to play a role in their decision. Many therapists answered that they base their decision on the extent of motor impairment (GMFM-88 in highly impaired children) and on the need for assessment with shoes and/or aids such as orthoses (GMFM-88). To a lesser extent the age of the child also influences the decision (GMFM-88 in young children). A few therapists seem to be inconsistent in their choice for a child, using the GMFM-88 only for their first assessment and continuing later with the GMFM-66.

Table 2 provides answers for the topic ‘goal and target-population’. Almost fifteen percent (8/54) of the therapists stated they use the GMFM-66 most frequently with a purpose other than evaluative. Additionally, the instrument is being used secondary as a diagnostic and prognostic tool by 23.5 % (8/34) and 67.6 % (23/34) of the therapists, respectively. All therapists indicated they use the GMFM-66 most frequently in patients with CP (53/53) and in patients between 5 months and 16 years of age (52/52). However, 62.7 % (32/51) of the therapists stated they also use the GMFM-66 in other populations, mainly those with acquired brain impairment, Down Syndrome and neuromuscular disorders. Twenty-four percent (12/50) of the therapists use the instrument in patients >16 years.

**Table 2:** Goal and target-population

Question	Frequency
Primary purpose	
Diagnostic	4/54 (7.4 %)
Evaluative	46/54 (85.2 %)
Prognostic	3/54 (5.6 %)
Else	1/54 (1.9 %)
Secondary purpose(s)	
Yes, namely	29/48 (60.4 %)
Diagnostic^a^	8/34 (23.5 %)
Evaluative^a^	6/34 (17.6 %)
Prognostic^a^	23/34 (67.6 %)
Else^a^	5/34 (14.7 %)
No	19/48 (39.6 %)
Primary population	
Cerebral Palsy	53/53 (100 %)
Else (e.g. Developmental Coordination Disorder, Neuromuscular disorders, Acquired brain impairment, Rheumatic disorders, Spina Bifida or Down Syndrome)	0/53 (0.0 %)
Secondary population(s)	
Yes	32/51 (62.7 %)
Cerebral Palsy^a^	1/35 (2.9 %)
Developmental Coordination Disorder^a^	0/35 (0.0 %)
Neuromuscular disorders^a^	8/35 (22.9 %)
Acquired brain impairment^a^	18/35 (51.4 %)
Rheumatic disorders^a^	1/35 (2.9 %)
Spina Bifida^a^	3/35 (8.6 %)
Down Syndrome^a^	13/35 (37.1 %)
Else^a^	10/35 (28.6 %)
No	19/51 (37.3 %)
Primary age category	
< 5 months	0/52 (0.0 %)
5 months – 5 years	19/52 (36.5 %)
6–11 years	29/52 (55.8 %)
12–16 years	4/52 (7.7 %)
> 16 years	0/52 (0.0 %)
Secondary age category	
Yes	46/50 (92.0 %)
< 5 months^a^	1/50 (2.0 %)
5 months – 5 years^a^	37/50 (74.0 %)
6–11 years^a^	19/50 (38.0 %)
12–16 years^a^	24/50 (48.0 %)
> 16 years^a^	12/50 (24.0 %)
No	4/50 (8.0 %)

^a^Multiple answers possible

Table 3 provides answers given to the topic ‘administration and scoring’. During administration 33.3 % (18/54) of the therapists indicated they use the English manual as a resource, while 16.7 % (9/54) use the Dutch manual of the GMFM-88 during administration of the GMFM-66 (expressed in the category ‘other’).

**Table 3:** Administration and scoring

Question	Frequency
Resources used during assessment	
User’s Manual^a^	18/54 (33.3 %)
English score form^a^	5/54 (9.3 %)
Dutch score form^a^	50/54 (92.6 %)
None of the above^a^	0/54 (0.0 %)
Else^a^	9/54 (16.7 %)
Number of items assessed per assessment	
1–12 items	0/54 (0.0 %)
13–24 items	6/54 (11.1 %)
25–36 items	8/54 (14.8 %)
37–48 items	6/54 (11.1 %)
49–60 items	7/54 (13.0 %)
61–65 items	4/54 (7.4 %)
Always all 66 items	23/54 (42.6 %)
Which items selected to be assessed	
Items expected to be succeeded	0/30 (0.0 %)
Items expected to be partly succeeded	7/30 (23.3 %)
Items expected not to be succeeded	1/30 (3.3 %)
Combination of the above	22/30 (73.3 %)
Number of sessions to assess the GMFM-66	
Always one session	11/54 (20.4 %)
Sometimes one session, sometimes more sessions	33/54 (61.1 %)
Always more sessions	10/54 (18.5 %)
Order of items assessed similar to order on score form	
Always similar order	13/52 (25.0 %)
Sometimes similar, sometimes different order	30/52 (57.7 %)
Always different order	9/52 (17.3 %)
Type(s) of instruction used	
Only verbal instruction	0/53 (0.0 %)
Only demonstration	0/53 (0.0 %)
Both above mentioned	49/53 (92.5 %)
Else	4/53 (7.5 %)
Usage of stimulation	
Stimulation if needed	50/51 (98.0 %)
Never stimulation	1/51 (2.0 %)
Providing help during assessment	
Always providing help	1/53 (1.9 %)
Sometimes providing help	24/53 (45.3 %)
Never providing help	28/53 (52.8 %)
Scoring based on	
Quality	4/52 (7.7 %)
Quantity	20/52 (38.5 %)
Combination of quality and quantity	23/52 (44.2 %)
Else	5/52 (9.6 %)
Number of trials per item	
Always 1 trial	2/53 (3.8 %)
Maximum of 2 trials	7/53 (13.2 %)
Maximum of 3 trials	40/53 (75.5 %)
Maximum of 4 trials	2/53 (3.8 %)
Maximum of 5 trials	1/53 (1.9 %)
Maximum of <5 trials	1/53 (1.9 %)
Procedure when being undecided between two scores	
Always the lowest score	36/50 (72.0 %)
Always the highest score	6/50 (12.0 %)
Else	8/50 (16.0 %)
Item score based on the trials	
Lowest score of all trials	1/51 (2.0 %)
Highest score of all trials	38/51 (74.5 %)
Modal score of all trials	8/51 (15.7 %)
Mean score	3/51 (5.9 %)
Else	1/51 (2.0 %)

^a^Multiple answers possible

None of the therapists indicated they administer less than 13 items per assessment. The therapists who answered that they always assess all 66 items were asked why they do so. The most mentioned reason was striving for completeness in order to get an overall picture of the child’s abilities. Some therapists answered that they do so to make entering scores in the GMAE possible. Respondents who stated ‘to assess a selection of items’ were asked which arguments they base their selection on. Foremost, therapists said they exclude items that they are convinced the child will definitely be able or definitely not be able to perform. Some therapists stated they focus on domains or items that are most relevant to the specific child and situation. Limited time also plays a role in the decision. Very few therapists answered to make use of item sets. Of those who responded to the open-ended question regarding clothing, almost half indicated they test children in their regular clothing, and one third said they demand something of the clothing such as for it to be comfortable. However, only a few therapists stated they remove clothes to observe children unobstructed or test children in particular clothing such as shorts and a t-shirt. Approximately half of the respondents stated they test children without shoes. Others test children ordinarily with shoes on, or with or without shoes depending on the child. Some therapists declared they test children without their aids/orthoses, while twice as many stated they test children with them.

Forty-seven percent (25/52) of the therapists indicated they sometimes or always provide help to the child during assessment. Almost forty percent (20/51) of the respondents scored solely based on quantity (extent of achievement of an item). One of the open-ended questions was: ‘A child refuses to attempt an item of which you expect him/her to (partially) succeed. How do you score this item?’. Approximately a quarter of respondents stated they make use of the ‘not tested’ (NT) approach. However, over one third answered they would rate it as 0. The minority said they would rate it by expectation, based on skills the child has shown during previous therapy sessions. Some therapists seem to be inconsistent, as the way they score an item that the child refuses varies between different children. Last, a frequently given solution was to repeat the item at a later moment. Seventy-five percent (40/53) of respondents said they provide the child with a maximum of 3 trials, 72.0 % (36/50) always use the lowest score when undecided between two scores for a trial, and 74.5 % (38/51) use the highest score of all trials.

Table 4 provides the answers given on the topic ‘interpretation’. Almost 30 % of the respondents (15/52) stated they calculate the total score of the GMFM-66 by the score form. The most frequently used function of the GMAE was the total score option, followed by case summary, item maps, CI, percentiles and SEM. On several open-ended questions some therapists gave comments from which it can be deduced they assume the GMAE to be expensive and for that reason do not use it. When the respondents were asked how they decide on the clinical meaning of the difference between the total scores of two tests, one third stated they compare the CI’s. Therapists also reported that they decide based on the graphical presentation given by the GMAE, percentiles and change on specific, relevant items. Some indicated they compare the total scores without explaining what constitutes a statistical difference. In addition, the answers show that the results of the GMFM-66 are being included in a broader perspective, for instance combined with the achievement of treatment goals. In response to the question regarding motivation for deviation from the guidelines provided by the manual, most therapists answered that they do not deviate from it. The few who did indicate they deviate mainly argued that they do so to adapt to the individual child or situation. A total of 14.0 % (7/50) of the respondents indicated they are interested in receiving the results of this study.

**Table 4:** Interpretation

Question	Frequency
Way of calculating total score	
By score form	15/52 (28.8 %)
By GMAE	37/52 (71.2 %)
Else	0/52 (0.0 %)
Functions of the GMAE being used	
No use of GMAE^a^	11/52 (21.2 %)
GMFM-66 total score^a^	41/52 (78.8 %)
Standard error of measurement^a^	24/52 (46.2 %)
Confidence interval^a^	28/52 (53.8 %)
Item maps^a^	29/52 (55.8 %)
Case summary^a^	30/52 (57.7 %)
Percentiles^a^	27/52 (51.9 %)
Else^a^	0/52 (0.0 %)

^a^Multiple answers possible

### Secondary analysis

Fourteen percent (4/28) of the therapists who attended the Training GMFM reported they use the GMFM-66 primarily for diagnostic purposes, and 28.6 % (6/21) secondarily for diagnostic purposes. Of the therapists who did not attend the training no one reported to use the GMFM-66 primarily for diagnostic purposes and 15.4 % (2/13) indicated they use it secondarily for diagnostic purposes. Almost 58 % (15/26) of therapists who did attend the Training GMFM responded they also use the GMFM-66 in non-CP patients, as opposed to 68.0 % (17/25) of therapists who did not attend the training.

Twenty-five percent (7/28) of the therapists who attended the training stated they use the manual while assessing the GMFM-66, while 42.3 % (11/26) of the therapists who did not attend the training stated they do. In response to a question on whether they scored the items based on quantity, quality or both, 44.4 % (12/27) of the therapists who attend the Training GMFM indicated they score items based on quantity. From the therapists who did not attend the training 32.0 % (8/25) did. Of therapists who did and did not attend the training 71.4 % (20/28) and 80.0 % (20/25) respectively reported that they score items based on three trials. Over twenty percent (21.4 %, 6/28) of therapists who did attend the training indicated that they score based on two trials. For the question ‘what is your procedure if you are undecided between two scores for a trial’ 85.2 % (23/27) of therapists who attended the training indicated they choose the lower score and 56.5 % (13/23) of therapists who did not attend the training answered the same way. The majority (85.2 %, 23/27) of therapists who attended the workshop define the item score based on the highest score of the trials. From the therapists who did not attend the training 62.5 % (15/24) do so.

Knowledge Brokers were compared to non-Knowledge Broker therapists. Over seventy percent (73.7 %, 14/19) of the Knowledge Brokers said they use the GMFM-66 secondarily in patients other than children with CP, as opposed to 54.8 % (17/31) of the therapists who are not Knowledge Brokers. Over one third of the Knowledge Brokers (31.6 %, 6/19) responded that they use the GMFM-66 secondarily in children aged >16 years. Twenty percent (6/30) of the therapists who are not Knowledge Brokers answered the same way.

Less than a quarter of the Knowledge Brokers (23,8 %, 5/21) stated that they use the manual while assessing the GMFM-66, while 40,6 % (13/32) of the therapists who are not Knowledge Brokers stated they use the manual. From the Knowledge Brokers and therapists who are not Knowledge Brokers 42.9 % (9/21) and 36.7 % (11/30) respectively indicated they score the items based on quantity. The majority of the Knowledge Brokers (81.0 %, 17/21) reported to score items based on three trials and 71.0 % (22/31) of the therapists who are not Knowledge Brokers similarly indicated this. For the question ‘how do you define the item score based on the performances on the different trials?’ 85.7 % (18/21) of the Knowledge Brokers indicated they base it on the highest score. From the therapists who are not Knowledge Brokers 69.0 % (20/29) did.

The results of the Fisher’s exact test indicate that respondents who attended the Training GMFM or are a Knowledge Broker do not use the GMFM-66 significantly more or less frequently in populations other than children with CP (respectively *χ*2 (1) = 0.58, *p* = 0.57 and *χ*2 (1) = 1.78, *p* = 0.24). However, these groups do seem to calculate the total score from the score form less frequently than respondents who did not attend the training or are not a Knowledge Broker (respectively *χ*2 (1) = 6.27, *p* = 0.02 and *χ*2 (1) = 5.76, *p* = 0.03). Cross-tabulations from the latter comparisons are presented in Table 5.

**Table 5:** Calculation of total score by ‘Attendance of Training GMFM’ and ‘Knowledge Broker’

		Calculation of total score		
		By score form	By GMAE	Total
Attendance of Training GMFM	Yes	4 (14.3 %)	24 (85.7 %)	28 (100 %)
	No	11 (45.8 %)	13 (54.2 %)	24 (100 %)
Total		15 (28.8 %)	37 (71.2 %)	52 (100 %)
Knowledge Broker	Yes	2 (9.5 %)	19 (90.5 %)	21 (100 %)
	No	12 (40.0 %)	18 (60.0 %)	30 (100 %)
Total		14 (27.5 %)	37 (72.5 %)	51 (100 %)

## Discussion

Overall, the therapists expressed a positive opinion of the GMFM-66. The user-friendly assessment and benefits of the GMAE were especially appreciated. The majority of questions pointed out that therapists deviate from the guidelines provided by the manual to a greater or lesser extent, with the high number of therapists who stated they calculate the total score of the GMFM-66 by the score form the most worrisome finding. Therapists who attended the Training GMFM and Knowledge Brokers act more in line with the guidelines on most issues, and calculate the total score significantly less frequently by the score form compared to therapists who did not attend the training respectively are not Knowledge Brokers.

The latter finding supports the conclusions of a study by Ketelaar et al., in which a substantial increase in therapists’ familiarity and confidence was observed one year after following a GMFM workshop. Although the current Training GMFM is not identical to the training evaluated by Ketelaar et al., the findings seem to demonstrate that the familiarity and confidence experienced by trained users is reflected in an increase in quality of application of the instrument. Also in accordance with our results, a previous study of Russell et al. found that 80 % of therapists involved in their study thought the GMFM-66 was useful for clinical purposes. However in that study 85 % of respondents indicated they would use item maps in clinical practice, while in our study 55.8 % declared they actually use item maps. These contrasting results can possibly be explained by recruitment for Russell et al.’s study taking place within centers involved in a CanChild research project, leading to a sample of evidence-based focused therapists motivated to use tools such as item maps.

The strong recruitment strategy of this study contributes to the generalizability of its results. One drawback of the strategy though is the overlap between therapists who were reached by different recruitment methods. As a result the exact number of therapists approached and response rate are unknown. Moreover, Knowledge Brokers were overrepresented in the study.

As a result of shortening and the addition of the GMAE, the GMFM-66 is often considered as an improved version of the GMFM-88. However, GMFM-66 is not merely an improvement of the GMFM-88, but an alternative with its own strengths, weaknesses and administration guidelines. Our study showed that therapists do not adequately recognize these differences. This results in strong deviations from the guidelines provided by the manual, and the risk of improper decision-making in pediatric rehabilitation increases. It also results in unjustified dissatisfaction with the GMFM-66. This can be seen in the comments made on the open-ended questions, specifically on general opinions of the test with and suggestions for improving it. Several problems that were highlighted by the physiotherapists are discussed in the manual, including using the GMFM-88 instead of the GMFM-66 for certain populations. Besides the GMFM-88 and GMFM-66 there are item sets available, including a specific selection of items based on a decision tree. There was no direct focus on item sets within this study. Nonetheless it should be mentioned that there was an inconsistency between the frequency of therapists mentioning the extensiveness of the GMFM-66, time needed to administer and using a selection of items, but almost no therapists mentioning the use of item sets in their answers.

Two-thirds of respondents indicated they use the GMFM-66 secondarily for prognostic purposes, despite the measure not being developed and tested for this purpose. However, motor growth curves of GMFM-66 scores stratified by severity (GMFCS-level) are available within the GMAE, based on a Canadian sample and additionally validated in a Dutch sample [8, 9]. The motor growth curves can be used to evaluate an individual’s gross motor function over time by comparing it to the average for their age/GMFCS-level and for goal-setting in rehabilitation. Although the motor growth curves provide some prognostic information, they should be handled with caution since within-stratum variation in motor development, based on other individual factors, is not taken into account in the development of the curves. Another possible explanation for therapists using the GMFM-66 for prognostic purposes is the availability of item maps. However, the Rasch analysis by which these were developed was based on a sample of Canadian children without validation in a Dutch sample. Furthermore, research has shown that therapists use cross-sectional percentiles by over-interpreting longitudinal comparisons. This is invalid since relatively large changes of percentile points are common [10] and Dutch validation is missing. To sum up, motor growth curves, item maps and percentiles can be of high value when they are used appropriately and their limitations are recognized. There is abundant room for further progress in individualizing predictive tools and validation of findings within Dutch populations.

The majority of therapists stated they use the GMFM-66 in populations other than children with CP. Hence additional validation of the GMFM-66 could fulfill the need for an appropriate gross motor function measure in other populations.

Given the way therapists administrate the GMFM-66 in clinical practice deviates to a large extent from the guidelines provided by the manual, information on psychometric properties and the previously described motor growth curves and percentiles, generated in highly controlled testing conditions, can at this moment not be generalized to clinical practice in the Netherlands.

The English manual is essential for in depth information on the administration guidelines of the GMFM-66, since no Dutch translation is available yet. However, less than half of the respondents used the English manual to increase their competency regarding the GMFM-66 and less than half used the English manual or Dutch GMFM-88 manual as a resource during assessment. Since the manuals are not user-friendly as a quick reference material, a concise Dutch factsheet including the most essential guidelines and the main differences between the GMFM-66 and GMFM-88 would be helpful for therapists in need for refreshment of their knowledge.

As stated earlier, the GMAE is required to calculate the total score of the GMFM-66, thus calculating total scores by the score forms is not valid. The finding that almost thirty percent of the therapists calculate total scores by the score form is therefore unexpected. Most likely these therapists use the GMFM-88 calculation on the score form to calculate the GMFM-66 total score. When one of the GMFM-66 assessments included in the manual is being calculated by the score form,^1^ this results in a score of 21 % (GMAE score 41.6, CI 43.1–47.2). Hence, two identical assessments can lead to approximately a doubling of the points, due to incorrect calculation of the total score. Such inaccuracy has extensive consequences. The rehabilitation team and parents may be misled regarding the development of the child. When decision-making in rehabilitation is based on incorrect conclusions the development of the child may be negatively influenced. Consequently, correct calculation of the GMFM-66 total score needs much more attention.

Only one third of the therapists stated they compare the two confidence intervals when deciding on the meaning of the difference between the total scores of two tests. Hence, it can be concluded that interpretation of the results of the GMFM-66 needs more attention. Therapists should be provided easy to use instructions for the comparison of two total scores. A useful function of the GMAE would be an automatic comparison of the CI’s of repeated measurements, including a conclusion of whether change is due to measurement error or true change. Additionally, more adequate and practical ways of interpreting change scores should be developed. First, the SEM, and thus the CI, is based only on the asymptotic error of the estimation process (how evenly the subjects are distributed around the score). The error of the assessment (e.g. by incorrectly recording a score on the score sheet) and the error of estimation (estimation of the GMFM-66 score from the responses to the items tested) are not included in the SEM [2]. An additional limitation is the SEM within the GMAE is currently based on a score instead of on test-retest parameters, which is not satisfying [11]. Hence, improvements are recommended with regard to the SEM. Moreover, minimal important change of the GMFM-66 should be defined.

## Conclusion

Overall therapists have positive opinions of the GMFM-66, particularly due to its user-friendly assessment and the benefits of the GMAE. For the majority of questions deviation from the guidelines provided by the manual was found to be occurring to a greater or lesser extent. Above all else the high number of therapists who stated that they calculate the total score of the GMFM-66 by the score form is worrisome. The consequences of the latter are far-reaching, since it has a misleading impact on the opinion of rehabilitation teams and parents on the development of the child, on decision-making in rehabilitation, and ultimately on the development of the child and quality of life of the family. Furthermore, we conclude motor growth curves, item maps and percentiles are of high value when used correctly. However, individualization of predictive tools and validation of findings within Dutch populations is necessary. Last, at this moment information on psychometric properties, motor growth curves and percentiles cannot be generalized to clinical practice in the Netherlands, as they are generated from highly controlled testing conditions, which do not hold in clinical practice.

## Additional file

Additional file 1: Figure S2. General opinion on the GMFM-66 and suggestions for improvement. (PDF 264 kb)

## Abbreviations

CI: Confidence interval
CP: Cerebral palsy
GMAE: Gross Motor Ability Estimator
GMFCS: Gross Motor Function Classification System
GMFM: Gross Motor Function Measure
GMFM-66: Gross Motor Function Measure-66
GMFM-88: Gross Motor Function Measure-88
NT: Not tested
NVFK: Nederlandse Vereniging voor Kinderfysiotherapie
In English: Dutch Association for Pediatric Physical Therapy
SEM: Standard error of measurement
WMO: Wet medisch-wetenschappelijk onderzoek met mensen
In English: Dutch Medical Research Involving Human Subjects Act

## Footnotes

1 Assessment of Colleen, score form on page 151–155, item map on page 163.
