# Supplementary material for: Application of the Gross Motor Function Measure-66 (GMFM-66) in Dutch clinical practice: a survey study
Source: BMC Pediatr. 2015 Oct 7;15:146. doi: 10.1186/s12887-015-0459-8 (PMC4597756; doi:10.1186/s12887-015-0459-8)
Supplement: Additional file 1: Figure S2. — General opinion on the GMFM-66 and suggestions for improvement. (PDF 264 kb) [file 12887_2015_459_MOESM1_ESM.pdf]

Integration in Fysioroadmap
Update of GMFM-training

General impression
- Positive
  - Useful
  - Clear
  - Nice

Application
- Useful for evaluative purpose

Content
- Disagreement on extent (short versus long)
- Items
  - Important items missing
  - Variable order appreciated
  - Order of items illogical
  - Too few on activity level
- Dimensions useful
- Item set appreciated

Shortening
Reorganize order of items

Assessment
- Very user friendly
- Difficult in children with mental retardation or behavioral issues
- Liked by children
- Disadvantage of testing without aids/orthoses
- Suitable environment needed
- Limited equipment needed

Instruction DVD
- More clear
- Examples of how to score performances

Space for comments behind each item score

Scoring
- Disagreement on objectivity / level of interpretability
- Accounts too little for compensating

More specific description of scoring descriptions
Dutch GMFM-66 manual

GMAE
- Valuable
- User friendly
- Useful options (percentiles / graphs / item maps)

Missing options
- Description of score-description per item
- Extension from motor growth curves
- More page-efficient printing options

Interpretation
- Limited perceived sensitivity to change
  - In general
  - Young children
  - Severely affected children
  - Slightly affected children (ceiling effect)
- Valuable for clinical practice (treatment plan)
- Comparable with clinical presentation
- Understandable for parents
- Videotaping useful

Reference tables
More responsive for severely affected children

General opinion

Suggestion
